# Structural Modifications of Bacterial Lipopolysaccharide that Facilitate Gram-Negative Bacteria Evasion of Host Innate Immunity

**DOI:** 10.3389/fimmu.2013.00109

**Published:** 2013-05-24

**Authors:** Motohiro Matsuura

**Affiliations:** ^1^Department of Microbiology, Graduate School of Medicine, Kyoto UniversityKyoto, Japan

**Keywords:** modification of lipopolysaccharide, less-acylated lipid A, innate immunity, immune evasion

## Abstract

Bacterial lipopolysaccharide (LPS), a cell wall component characteristic of Gram-negative bacteria, is a representative pathogen-associated molecular pattern that allows mammalian cells to recognize bacterial invasion and trigger innate immune responses. The polysaccharide moiety of LPS primary plays protective roles for bacteria such as prevention from complement attacks or camouflage with common host carbohydrate residues. The lipid moiety, termed lipid A, is recognized by the Toll-like receptor 4 (TLR4)/MD-2 complex, which transduces signals for activation of host innate immunity. The basic structure of lipid A is a glucosamine disaccharide substituted by phosphate groups and acyl groups. Lipid A with six acyl groups (hexa-acylated form) has been indicated to be a strong stimulator of the TLR4/MD-2 complex. This type of lipid A is conserved among a wide variety of Gram-negative bacteria, and those bacteria are easily recognized by host cells for activation of defensive innate immune responses. Modifications of the lipid A structure to less-acylated forms have been observed in some bacterial species, and those forms are poor stimulators of the TLR4/MD-2 complex. Such modifications are thought to facilitate bacterial evasion of host innate immunity, thereby enhancing pathogenicity. This hypothesis is supported by studies of *Yersinia pestis* LPS, which contains hexa-acylated lipid A when the bacterium grows at 27°C (the temperature of the vector flea), and shifts to contain less-acylated forms when grown at the human body temperature of 37°C. This alteration of lipid A forms following transmission of *Y. pestis* from fleas to humans contributes predominantly to the virulence of this bacterium over other virulence factors. A similar role for less-acylated lipid A forms has been indicated in some other bacterial species, such as *Francisella tularensis*, *Helicobacter pylori*, and *Porphyromonas gingivalis*, and further studies to explore this concept are expected.

## Pattern Recognition of Microbial Components

**Figure 1:**
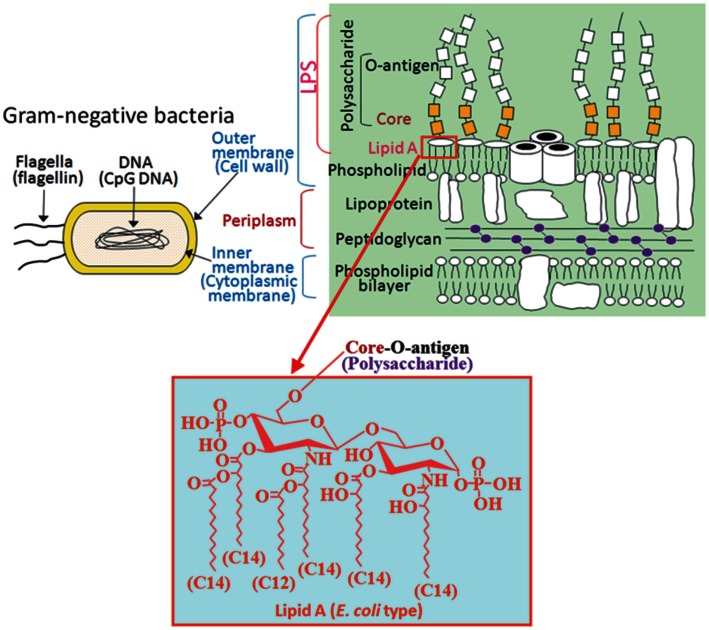
**PAMPs included in Gram-negative bacteria**. Flagellin and CpG-DNA recognized by TLP5 and TLR9, respectively, are found not only in Gram-negative bacteria but also in Gram-positive bacteria. On the other hand, LPS recognized by TLR4 is found only in Gram-negative bacteria as a cell wall component. A hydrophobic membrane anchor portion of LPS termed lipid A, but not the polysaccharide portion, is responsible for stimulation of TLR4 signaling. *E. coli* type hexa-acylated lipid A, relatively conserved among a wide variety of Gram-negative bacteria, is the most potent structure that activates the TLR4 pathway.

The invasion of microorganisms into mammalian hosts is initially sensed by phagocytic cells through their receptors, known as pattern-recognition receptors (PRRs), to activate the innate immune response, which is the first line of host defense against pathogens. Each PRR can recognize a group of microbial components having a similar structural pattern, termed the pathogen-associated molecular pattern (PAMP), and a limited number of PRRs are enough for surveillance of almost all microbial pathogens. The toll-like receptor (TLR) family is a representative PRR family, and different members in this family react with specific PAMPs (Table 1). In Gram-negative bacteria, PAMPs such as flagellin, CpG-DNA, and lipopolysaccharide (LPS) are involved (Figure 1), although flagellin and CpG-DNA are not limited to Gram-negative bacteria. Flagellin is a protein contained in bacterial flagella observed mainly in motile bacteria independent of Gram-negative or -positive status. CpG-DNA is a term for unmethylated CpG dinucleotides in a particular base context that is abundant in bacterial genomic DNA, but rare in mammalian genomes, and is generally found in both Gram-negative and -positive bacteria. On the other hand, LPS is a cell wall component characteristic of Gram-negative bacteria (Figure 1), but not of Gram-positive bacteria, and is generally the most potent immunostimulant among bacterial cell wall components. For defense against Gram-negative infections in general, LPS is the most suitable target PAMP for mammalian host cells.

**Table 1:** **PAMPs recognized by the toll-like receptors (TLRs)**.

TLRs	PAMPs	Origin of PAMPs	Reference
TLR1/2	Tri-acylated lipopeptide	Bacteria, mycoplasma	Takeuchi et al. (2002)
TLR2	Lipoprotein/lipopeptide Peptidoglycan Lipoteichoic acid	Gram-positive bacteria	Aliprantis et al. (1999), Takeuchi et al. (2000), Echchannaoui et al. (2002)
TLR3	Double-stranded RNA	Virus	Edelmann et al. (2004)
TLR4	LPS	Gram-negative bacteria	Poltorak et al. (1998), Chow et al. (1999), Hoshino et al. (1999), Qureshi et al. (1999)
TLR5	Flagellin	Flagellated bacteria	Hayashi et al. (2001), Hawn et al. (2003)
TLR6/2	Diacyl lipopeptide	Mycoplasma	Takeuchi et al. (2001)
TLR7	Single-stranded RNA	RNA virus	Kumar et al. (2009)
TLR8	Single-stranded RNA	RNA virus	Kumar et al. (2009)
TLR9	Unmethylated CpG-DNA	Bacteria DNA virus	Hemmi et al. (2000), Krieg (2002)
			Lund et al. (2003), Hochrein et al. (2004), Krug et al. (2004a,b), Tabeta et al. (2004)

## Structure and Activity of LPS Constituents

Lipopolysaccharide consists of a hydrophobic membrane anchor portion known as lipid A and a non-repeating core oligosaccharide coupled to a distal polysaccharide (O-antigen) that extends from the bacterial surface (Raetz and Whitfield, 2002). Within this class of substances, there is an enormous multitude of natural structural variants that are primarily due to the extended diversity in chemical composition of the polysaccharide region (core and O-antigen), but also due to considerable variations in the fine structure of lipid A (Raetz and Whitfield, 2002). In LPS from most Gram-negative bacteria, the O-specific chain consists of up to 50 repeating oligosaccharide units formed of 2–8 monosaccharide components in a highly species- and strain-specific manner. In the vast majority of LPS structures, the O-specific chain is characterized by extremely high structural variability even within a given bacterial species, which constitutes the chemical basis for the serological classification of individual wild-type bacterial strains according to their O-antigenic determinants. Bacterial mutants having LPS without O-specific chains are able to grow and multiply *in vitro*, showing that the O-chain in principle is dispensable for bacterial viability. Based on characteristic colony morphology, distinct from the smooth (S)-form of wild-type enterobacterial species, these mutants have been termed as rough (R)-mutants leading to a corresponding general sub-classification into S- and R-form LPS. When LPS molecules extracted from any S-LPS-containing strain are separated by SDS-PAGE, there is extensive heterogeneity in the size of the molecules due to variations in the chain length of the O-polysaccharides. This is evident in the classical “ladder” pattern in SDS-PAGE, where each “rung” up the ladder represents a lipid A-core molecule substituted with an increment of one additional O-unit. The spacing between the rungs is determined by the size of the O-unit.

The primary role(s) of the O-polysaccharides appears to be protection for bacteria. In animal pathogens, O-polysaccharides may contribute to bacterial evasion of host immune responses, particularly the alternative complement cascade. Assembly of the membrane attack complex is affected by the chemistry of the O-polysaccharide, its chain length, and the relative amounts of long chain S-LPS (Rautemaa and Meri, 1999; Murray et al., 2006). In addition, LPS from some bacteria, such as *Helicobacter pylori, Neisseria gonorrhoeae, N. meningitidis*, and *Haemophilus influenzae*, have been found to contain O-antigen structures that closely resemble human glycosphingolipids due to the presence of common host carbohydrate residues such as *N*-acetylneuraminic acid or l-fucose (Moran et al., 1996). For example, *H. pylori*, a prevalent gastroduodenal pathogen of humans, produces LPS O-antigen units that can be uniquely decorated by the addition of fucose residues to generate Lewis antigens, carbohydrates that are also expressed by the gastric epithelium in humans. Lewis x (Le^x^) and Lewis y (Le^y^) are the dominant Lewis antigens in *H. pylori* LPS. Le^x^ is synthesized by the addition of a fucose residue to *N*-acetyl-β-lactosamine (LacNAc) units in the O-antigen chains, and di-fucosylated Le^y^ is synthesized by the addition of a second fucose residue to Le^x^. A typical *H. pylori* O-antigen chain is glycosylated with multiple internal Le^x^ units and possesses either Le^x^ or Le^y^ at the terminal position. It has been observed that disease-associated isolates of *H. pylori* show a higher tendency to present LPS that are glycosylated with both Le^x^ and Le^y^, while the LPS of isolates obtained from normal mucosa generally present either Le^x^ or Le^y^ exclusively, or neither (Skoglund et al., 2009). These structures highly decorated with Lewis antigens have been suggested to be important for gastric colonization, adhesion, and immune evasion through molecular mimicry where the Lewis antigens provide a “camouflage” for the bacteria in order to escape the host innate immune response (Moran, 2008).

**Figure 2:**
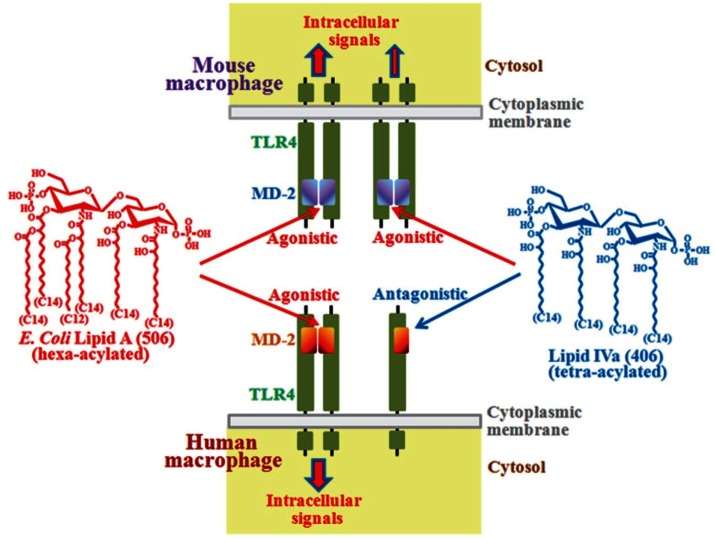
**Differential recognition of lipid A structures between human and mouse TLR4/MD-2 complexes**. TLR4 is a type I transmembrane molecule, and MD-2 is an extracellular molecule that associates with the extracellular region of TLR4. Lipid A can bind to MD-2 but not to TLR4. Binding of lipid A to MD-2 induces dimerization of the TLR4/MD-2 complex for transduction of stimulatory signals into cells. Recognition of lipid A structures by mouse MD-2 and human MD-2 is different. For example, *E. coli* type hexa-acylated lipid A is recognized as a strong agonist by both mouse and human MD-2 that causes dimer formation. On the other hand, tetra-acylated lipid IVa can bind to both types of MD-2, but subsequent dimer formation is achieved only by the mouse system and not by the human system. Once this structure binds to human MD-2, dimer formation is suppressed, and as a result, this structure acts as an antagonist.

The core oligosaccharide has much less structural variation compared to the hypervariable O-polysaccharides and has limited variation within a given bacterial genus. Neither the O-antigen nor the core oligosaccharide is required for the immunostimulatory activity of LPS, but rather the lipid A portion is responsible for the activity. A representative type of lipid A is seen in *Escherichia coli*, the structure of which is a β-1→6-linked glucosamine disaccharide phosphorylated at the 1 and 4′ positions and acylated with 6 acyl groups of 12–14 carbon chain lengths (Figures 1 and 2). This particular type of hexa-acylated lipid A elicits robust immunostimulatory activity.

Although not secreted by bacterial cells, small amounts of the LPS are liberated into the medium under some circumstances such as during cell division. Larger amounts are released by bacteria killed by antibiotics, phagocytosis, the complement complex, or treatment with divalent cation chelators. In an infected host, small amounts of LPS can be protective by stimulating the immune system, while large amounts induce high fever and lead to septic shock and death by multiorgan failure and systemic inflammatory response. LPS liberated from bacteria associates with LPS binding protein (LBP), an acute phase protein present in the bloodstream (Schumann et al., 1990), and forms complexes consisting of LPS, LBP, and soluble CD14 (sCD14) (Tobias et al., 1995). CD14 is either present in circulation as a sCD14 or is expressed on the surface of phagocytes as a glycosylphosphatidylinositol (GPI)-anchored molecule (membrane CD14, mCD14) (Wright et al., 1990). LPS is delivered from the complexes to mCD14 on the cell surface and then transferred to MD-2 for LPS signaling. CD14 does not appear to be essential for LPS responses, but probably has a role in their amplification (Haziot et al., 1998).

## Species Differences in Recognition of Lipid A Structures

Signals for the immunostimulatory activity are transduced into cells after interaction of LPS (lipid A moiety) with the TLR4/MD-2 receptor complex that is expressed on the surface membrane of mammalian cells (Miyake et al., 2000; Miyake, 2004) (Figure 2). TLR4 is a type I transmembrane molecule containing a large leucine-rich repeat in the extracellular region where the MD-2 molecule associates. The extracellular domain of TLR4 is not sufficient for LPS recognition, but MD-2 is essential for the recognition (Shimazu et al., 1999; Akashi et al., 2000; Schromm et al., 2001; Nagai et al., 2002). MD-2 is a ligand-binding component of the TLR4/MD-2 complex (Shimazu et al., 1999; Akashi et al., 2000; Schromm et al., 2001; Nagai et al., 2002), and acyl groups of lipid A are buried inside a unique hydrophobic cavity of MD-2 (Park et al., 2009). Binding of lipid A to MD-2 induces dimerization of TLR4/MD-2 by altering the conformation of the Phe126 surface of MD-2 and exposing otherwise hidden interaction sites for binding to the C-terminal domain of the second TLR4 molecule. It has been suggested that dimerization of the extracellular domains of TLR4 leads to proper orientation of the intracellular TIR domains, recruitment of adaptor proteins, and initiation of intracellular signaling (Jin and Lee, 2008) (Figure 2).

In naturally isolated lipid A preparations, heterogeneous lipidic materials and minor contaminants that may influence the activity are involved. To avoid such possible influences, chemically synthesized diverse lipid A structures are used for studies of structure-activity relationships, and the lipid A structure of the *E. coli* type (Figure 2) has been found to be the most potent immunostimulator (Galanos et al., 1985; Homma et al., 1985; Kotani et al., 1985). These studies indicate that the number and carbon chain length of acyl groups attached to the phosphorylated glucosamine backbone are critical for TLR4 activation, and that alteration of these factors can reduce the magnitude of the activation (Schromm et al., 2000; Kusumoto et al., 2003). Moreover, it has also been found that the response of human cells to such altered structures is stricter than that of mouse cells (Golenbock et al., 1991; Matsuura et al., 1999). For example, *E. coli* type hexa-acylated lipid A (synthetic compound 506) acts as a strong TLR4 agonist to both human and mouse macrophages, while tetra-acylated lipid IVa (synthetic compound 406) acts as an agonist to mouse macrophages, although the activity is weaker than that of compound 506, and as an antagonist (suppressor to agonist) to human macrophages (Figure 2). Lipid IVa is a structure identified as a biosynthetic precursor of lipid A. Lipid IVa binds to both mouse and human MD-2, whereas it induces dimerization of the mouse TLR4/MD-2 complex, but not of the human TLR4/MD-2 complex (Akashi et al., 2001; Saitoh et al., 2004). Some amino acid residues are not conserved between human and mouse TLR4/MD-2 molecules. These include amino acid residues of MD-2, like the hydrophobic residues (Leu125 and Pro127) in the Phe126 loop of mouse MD-2 (corresponding to human Lys125 and Ser127, respectively), and amino acids of TLR4, like Lys367 and Arg434 located near the dimerization interface of mouse TLR4 (corresponding to human Glu369 and Gln436, respectively), that are suggested to participate in the dimerization of mouse TLR4/MD-2 mediated by lipid IVa (Ohto et al., 2012).

## Variation of Lipid A Structures

Among natural lipid A, the hexa-acylated *E. coli* type is relatively conserved in a wide variety of Gram-negative bacteria, although some bacterial species have different types of lipid A. Even in a single species of bacteria, some variants of lipid A frequently coexist and their structures are sometimes modified under different environmental conditions (Raetz and Whitfield, 2002). A variety of human pathogens, including *Yersinia pestis* and *Francisella tularensis*, have lipid A moieties that are poorly recognized by the human TLR4/MD-2 complex (Kawahara et al., 2002; Vinogradov et al., 2002; Phillips et al., 2004). These lipid A species typically consist of only four or five acyl groups, some of which are 16–18 carbons in length. It is known that LPS-hyporesponsive mice (C3H/HeJ), which have a dysfunctional mutant TLR4, are highly susceptible to infection with Gram-negative bacteria (O’Brien et al., 1982; Vogel et al., 1999). This implies that the potential for those pathogens to cause severe disease in humans is likely attributable to their weak lipid A activity for TLR4 signaling.

## Virulence Factors in Relation to *Y. pestis* Infection

*Y. pestis* is the causative agent of plague in humans and is primarily a rodent pathogen that is transmitted to humans through the bite of an infected flea (Perry and Fetherston, 1997). The temperature range for a flea residing in rodent burrows or mammalian hair is around 25°C, while the body temperature of rodents and humans is around 37°C. In the infection cycle of *Y. pestis*, this bacterium survives in two different temperature environments in which various bacterial cellular components are differently expressed (Brubaker, 1991; Straley and Perry, 1995; Anisimov et al., 2004). The production of several virulence factors, such as the fraction 1 antigen (Du et al., 2002), the pH 6 antigen (Huang and Lindler, 2004), Yop proteins (Viboud and Bliska, 2005), and the type III secretion system (Cornelis, 2002), are upregulated during growth of the bacterium at 37°C. In contrast, the production of murine toxin (Hinnebusch et al., 2002), which is required for the survival of the bacterium in the midgut of fleas, is synthesized at 27°C and is downregulated at 37°C. Structural changes of lipid A in the bacterium between those two different temperature ranges have also been found based on MALDI-TOF mass spectrometric analysis (Kawahara et al., 2002). The lipid A present in LPS of the bacterium is heterogeneous (from hexa- to tri-acylated types) when it is grown at 27°C, and shifts to the hypo-acylated types (tetra- and tri-acylated types) when it is grown at 37°C. LPS isolated from *Y. pestis* grown at 37°C has been revealed to exhibit much weaker stimulation activity of human macrophages for proinflammatory cytokine production than that from the bacterium grown at 27°C (Montminy et al., 2006; Matsuura et al., 2010). Such a shift of lipid A structures to hypo-acylated types probably weakens the bacterial ability to stimulate TLR4 signaling and facilitates bacterial evasion of host innate immunity, allowing free growth in the host and thereby enhancing bacterial virulence. This infection strategy of *Y. pestis* seems to play an important role, since the bacteria need to achieve remarkable multiplication rates after inoculation of only a few bacteria in the skin delivered by a flea bite to develop bubonic plague. A genetically modified strain of *Y. pestis* (KIM5-pLpxL strain) expressing a potent TLR4-activating hexa-acylated lipid A at 37°C has been prepared, and the strain is unable to cause systemic disease in wild-type mice, despite the presence of other well-established virulence factors (Montminy et al., 2006). In addition, the administration of TLR4 agonists has been demonstrated to augment host defense against lethally high doses of *Y. pestis* in a mouse pneumonic plague model (Airhart et al., 2008). These studies indicate that the ability to evade TLR4 activation by lipid A alteration is critical for *Y. pestis* virulence.

## Role of Lipid A Alteration During *Y. pestis* Infection

**Figure 3:**
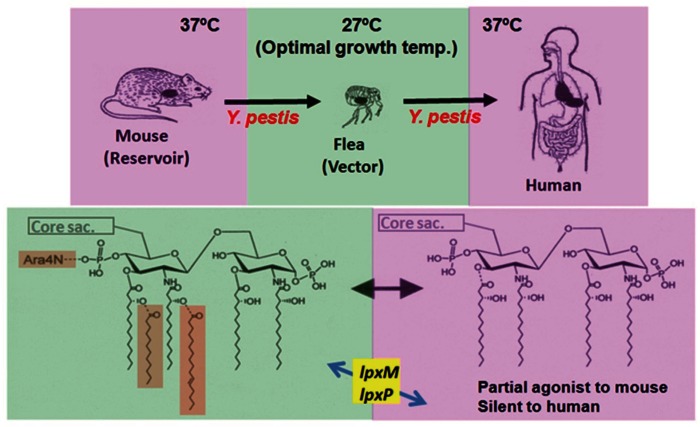
**Infection cycle of *Y. pestis* and temperature-dependent alteration of its lipid A structures**. *Y. pestis*, a causative agent of plague, grows in mice at 37°C by possessing a tetra-acylated type as its major lipid A species. This lipid A species acts as a partial agonist to mouse cells, and *Y. pestis* is recognized by the mouse innate immune system to some extent but not enough to eliminate it completely. As a result, moderate amounts of the bacteria can survive in mice for a prolonged period, and infected mice serve as a reservoir of *Y. pestis*. Through flea bites, this bacterium moves into fleas and grows actively at 27°C, its optimal growth temperature. At this temperature, expression of the late acyltransferase genes (*lpxM* and *lpxP*) is upregulated, and a hexa-acylated type becomes predominant among its lipid A species. Then, the bacterium moves into a human body through the bite of an infected flea and grows at 37°C. At this temperature, the expression of the late acyltransferase genes is downregulated, and the major lipid A species shift to the tetra-acylated type. *Y. pestis* containing such lipid A species is not sensed (is silent) by the human TLR4-mediated innate immune system, and the bacteria can grow freely and induce severe diseases.

As mentioned above, the scheme summarized in Figure 2 was obtained from an analysis of the structure-activity relationship using chemically synthesized lipid A analogs. In studies of *Y. pestis*, LPS preparations isolated from bacteria grown at 27 or 37°C by the conventional phenol-water extraction method are used. Chemical analysis of these natural LPS preparations reveals that LPS at 27°C contains a mixture of lipid A from hexa- to tri-acylated types, and LPS at 37°C a mixture of only tetra- and tri-acylated types (Kawahara et al., 2002). The abilities of such LPS preparations to stimulate mouse and human macrophages have been investigated, and results clearly show that LPS at 27°C exhibits strong agonistic activity to both mouse and human macrophages, while LPS at 37°C exhibits agonistic activity to mouse macrophages (although weaker than that of LPS at 27°C), but antagonistic activity to human macrophages (Matsuura et al., 2010). These results indicate that LPS at 27°C and LPS at 37°C behave just like hexa-acylated lipid A and tetra-acylated lipid A as shown in Figure 2, respectively. It is believed that LPS is not easily released from bacterial cell walls without lysis or the destruction of bacterial cells, and the behavior of bacterium-bound LPS rather than bacterium-free LPS is considered to be more important for investigating the role of LPS during bacterial infections. To evaluate the role of bacterium-bound LPS, formalin-killed bacteria (FKB) have been prepared from *Y. pestis* grown at 27 or 37°C, and their ability to stimulate human macrophages has been examined (Matsuura et al., 2010). FKB grown at 27°C strongly stimulates human macrophages for proinflammatory cytokine production, similar to LPS at 27°C. This activity is substantially suppressed in the presence of an anti-TLR4 antibody, indicating that the LPS-TLR4 signaling pathway plays a dominant role in reacting to whole bacterial cells among the pathways mediated by various bacterial cell components. In contrast, FKB grown at 37°C shows no antagonistic activity, unlike LPS at 37°C, but instead exhibits weak agonistic activity that is probably dependent on bacterial components other than LPS. This suggests that for antagonistic activity, but not for agonistic activity, LPS is required to be free from bacterial cells. Taken together, the important role of lipid A alteration in the infection cycle of *Y. pestis* is understood as summarized in Figure 3. *Y. pestis* grown at 37°C has LPS-containing hypo-acylated lipid A species that act as partial agonists to mouse cells, but act as neither agonists nor antagonists to human cells. Upon infection with *Y. pestis*, the mouse innate immune system responds to some extent, but not so strongly as to completely eliminate the bacteria, leading to the prolonged presence of a moderate amount of bacteria. As a result, the mouse is a reservoir of *Y. pestis*. On the other hand, the human innate immune system cannot respond to the bacteria at all, even though TLR4 signaling is not suppressed. As a result, *Y. pestis* grows freely in the human body and induces severe diseases such as bubonic, septicemic, and pneumonic plague. Recently, transgenic mice expressing human rather than mouse TLR4/MD-2 have been generated to test whether the blunted recognition of hypo-acylated LPS by the human receptor complex dictates susceptibility to infection of *Y. pestis* (Hajjar et al., 2012). These “humanized” mice are indeed more sensitive to *Y. pestis* infection than wild-type mice, supporting the idea that evasion of recognition by TLR4/MD-2 promotes *Y. pestis* virulence in humans.

## Pathogenic *Yersinia* and Lipid A

The biosynthesis of lipid A and its modification has been intensively studied using *E. coli* (Raetz et al., 2007). The final steps of lipid A synthesis occur in the inner membrane, where two acyl groups are added to the tetra-acylated keto-deoxyoctulosonate (Kdo)-lipid IVa before the mature hexa-acylated lipid A is exported to the outer membrane. Kdo is a sugar component found in all known core oligosaccharide species of LPS. The late acyltransferases HtrB (LpxL) and MsbB (LpxM) consecutively add lauroyl (C_12_) and myristoyl (C_14_) groups to the tetra-acylated intermediate (Clementz et al., 1996, 1997; Somerville et al., 1996). In *Y. pestis*, homologs of the late acyltransferase genes, *lpxP* and *lpxM*, have been identified (Rebeil et al., 2006). LpxP acts instead of LpxL to add a palmitoleate (C_16:1_) to the 3-hydroxy myristoyl (3-OH C_14_) group substituting at the 2′ position of the glucosamine disaccharide backbone. Expression of these genes in *Y. pestis* has been revealed to be suppressed at 37°C while upregulated at 27°C (Figure 3). Through this study, the underlying mechanism for temperature-dependent alteration of the *Y. pestis* lipid A structure has been clarified. In addition to *Y. pestis*, two more important human pathogens are included in the genus *Yersinia*, *Y. pseudotuberculosis*, and *Y. enterocolitica*. These three species of bacteria have been cultured at 21 and 37°C to analyze the structural differences of the lipid A forms between them (Rebeil et al., 2004). After growth at 37°C, each of these species synthesizes LPS-containing mainly tetra-acylated forms of lipid A, and shifts to increased acylation of lipid A to hexa-acylated forms at 21°C, although some differences are seen in the number and type of acyl groups between each species. Moreover, LPS preparations from these species grown at 21°C have been confirmed to strongly stimulate human cells, while those at 37°C do not. In contrast to *Y. pestis*, which causes highly invasive and usually lethal infections, *Y. pseudotuberculosis* and *Y. enterocolitica* cause relatively mild food- and water-borne gastroenteritis; however, the temperature changes during their infection cycles from lower temperatures (external environment or flea) to higher temperatures (mammalian body) are similar. These results suggest that the production of a less immunostimulatory form of LPS upon entry into the mammalian host is a conserved pathogenesis mechanism in the genus *Yersinia*.

## Bacteria with Less-Acylated Lipid A

*Francisella tularensis*, a causative agent of zoonotic tularemia, contains LPS with less-acylated lipid A. The major lipid A molecule in this bacterium is reported to be a monophosphoryl tetra-acylated lipid A with three 3-OH C_18_ acyl groups and one C_16_ acyl group (Vinogradov et al., 2002; Phillips et al., 2004). LPS isolated from this bacterium shows neither agonistic activity nor antagonistic activity to TLR4 signaling in either human or mouse cells (Hajjar et al., 2006). These so-called silent characteristics of *F. tularensis* LPS are thought to contribute to its capacity to evade mammalian immune defense mechanisms and to promote survival in an infected host (Sjostedt, 2006; Gallagher et al., 2007). The effect of the preventive administration of a synthetic TLR4 agonist on the protection of mice from experimental pneumonic tularemia has been demonstrated (Lembo et al., 2008). Considering this result, together with the protective effect of TLR4 agonists against lethal infection of *Y. pestis* in a mouse model (as mentioned above), prophylactic administration of TLR4 stimulating agents may be useful for the prevention of infectious diseases caused by pathogens that evade TLR4-mediated host innate immunity.

*H. pylori* produces LPS with unique structures, not only in its O-antigen region (as mentioned above) but also in its lipid A region. It is well known that *H. pylori* lipid A shows up to 1,000-fold weaker activity when stimulating TLR4 than does *E. coli* lipid A (Mandell et al., 2004). Therefore, it is thought that the LPS structure of *H. pylori* has evolved to aid the bacterium in evading the host innate immune system, thereby contributing to chronic infection. The major lipid A species in LPS of this bacterium is a tetra-acylated type; however, the process to produce this lipid A species is different from that of *Y. pestis*. In *H. pylori*, the hexa-acylated lipid A species is first synthesized, and then several modification enzymes, including a deacylase, function to produce the tetra-acylated lipid A species (Stead et al., 2008).

*Porphyromonas gingivalis* is considered to be an important agent in human periodontal diseases. The LPS of this bacterium contains highly variant lipid A species: in addition to di- and mono-phosphorylated and penta- and tetra-acylated species, non-phosphorylated species are also present (Rangarajan et al., 2008; Coats et al., 2009). It is believed that the LPS of this bacterium, which contains various lipid A species with weak agonistic and strong antagonistic activities to TLR4 signaling, is a critical virulence factor to evade the host innate defense system.

## Contribution of Lipid A Alteration to Live Bacterial Reactions

Most of the above mentioned studies have investigated the activity of isolated LPS preparations having less-acylated lipid A species. However, the contribution of their activity to the direct interaction of live bacteria with host mammalian cells, especially with human cells, during infection has not been well studied. To address this issue, mutant strains of *Salmonella enterica* serovar typhimurium (*S. typhimurium*) having fewer lipid A acyl groups (penta- and/or tetra-acylated types) have been established. These mutants have been used to infect human cells to investigate their stimulatory activity (Matsuura et al., 2012). The stimulatory activity of live bacteria on human cells disappears suddenly when only one acyl group of lipid A is eliminated from that of the wild-type strain, from a hexa- to a penta-acylated lipid A type. The antagonistic activity on TLR4 signaling observed in less-acylated LPS disappears in the live bacterial reaction, as is the case with FKB of *Y. pestis* grown at 37°C, indicating that the antagonistic activity of LPS is unable to be displayed in whole cell reactions. This study demonstrated that the important role of less-acylated lipid A in live bacteria is to present a silent, but not antagonistic, state to TLR4 signaling.

## Modified Lipid A in Clinical Isolates

It has been found that a surprisingly large fraction of meningococcal isolates have LPS with under-acylated (penta-acylated) lipid A as a result of a mutation of the *lpxL* gene (Fransen et al., 2009), which likely arises spontaneously in hosts. This finding revealed the important pathogenic role of lipid A modification in a particular infectious disease. Findings of similar LPS mutants among clinical isolates of other infectious diseases are expected, since such investigations have rarely been carried out before now.

## Future Perspectives

Data are accumulating to support the idea that modification of lipid A structures to less-acylated species leads to bacterial evasion of host innate immunity. However, most of these studies have been carried out by using chemically synthesized compounds or isolated LPS preparations, but usually not with whole bacterial cells, especially live bacteria. The data indicating differential behavior of bacterium-bound LPS with less-acylated lipid A compared to bacterium-free LPS, and that show a dominant role of the LPS-TLR4 signaling pathway among the pathways mediated by various bacterial cell components, have been obtained from experiments using whole bacteria as described above. LPS-altering mutants can be associated with changes in other bacterial virulence determinants, and it is important to understand how these factors interact with each other to cause detrimental effects on the host in practical disease situations. Infection experiments using living bacteria will improve our understanding of the underlying mechanisms of how other factors associate with lipid A alterations in the induction of infectious diseases.

Species differences in the specificity of the interaction of TLR4/MD-2 with LPS prevent generalization to humans from mouse disease models, the most extensive sources of evidence to date. To overcome this problem, transgenic mice expressing human TLR4/MD-2 have been generated recently, as described above. Such humanized mice, not only for TLR4/MD-2 but also for other molecules that participate in infectious diseases, are potentially strong tools for practically analyzing the roles of lipid A alteration in human infectious diseases. For the development of protective and therapeutic agents against infectious diseases, humanized mice can also be used to test efficacy. In particular, when evaluating the safety of TLR4 agonistic agents, humanized mice may play an important role since humans are more sensitive to the endotoxic activity of LPS than are mice.

## Conflict of Interest Statement

The authors declare that the research was conducted in the absence of any commercial or financial relationships that could be construed as a potential conflict of interest.
